# A computational application for multi-skill nurse staffing in hospital units

**DOI:** 10.1186/s12911-018-0638-2

**Published:** 2018-06-28

**Authors:** Ana Respicio, Margarida Moz, Margarida Vaz Pato, Rute Somensi, Cecília Dias Flores

**Affiliations:** 1 CMAF-CIO, Faculdade de Ciências, Universidade de Lisboa, Lisbon, Portugal; 2 ISEG and CMAF-CIO, Universidade de Lisboa, Lisbon, Portugal; 3 Pavilhão Pereira Filho, Santa Casa de Misericórdia Porto Alegre and Universidade Federal de Ciências da Saúde de Porto Alegre, Hospital São José, Porto Alegre, Brazil; 4 Universidade Federal de Ciências da Saúde de Porto Alegre, Porto Alegre, Brazil; 5 Departamento de Informática, Faculdade de Ciências, Universidade de Lisboa, Bloco C6, Piso 3, 1749-016 Lisboa, Portugal

**Keywords:** Nurse staffing, Multi-skill staffing, Shift work, Spreadsheet staffing model, Decision support

## Abstract

**Background:**

Approaches to nurse staffing are commonly concerned with determining the minimum number of care hours according to the illness severity of patients. However, there is a gap in the literature considering multi-skill and multi-shift nurse staffing. This study addresses nurse staffing per skill category, at a strategical decision level, by considering the organization of work in shifts and coping with variability in demand.

**Methods:**

We developed a method to determine the nursing staff levels in a hospital, given the required patient assistance. This method relies on a new mathematical model for complying with the legislation and guidelines while minimizing salary costs. A spreadsheet-based tool was developed to embed the model and to allow simulating different scenarios and evaluating the impact of demand fluctuations, thus supporting decision-making on staff dimensioning.

**Results:**

Experiments were carried out considering real data from a Brazilian hospital unit. The results obtained by the model support the current total staff level in the unit under study. However, the distribution of staff among different skill categories revealed that the current real situation can be improved.

**Conclusions:**

The method allows the determining of staff level per shift and skill depending on the mix of patients’ illness severity. Hospital management is offered the possibility of optimizing the staff level using a spreadsheet, a tool most managers are familiar with. In addition, it is possible to evaluate the implications of decisions on workforce dimensioning by simulating different demand scenarios. This tool can be easily adapted to other hospitals, using local rules and legislation.

**Electronic supplementary material:**

The online version of this article (10.1186/s12911-018-0638-2) contains supplementary material, which is available to authorized users.

## Background

Healthcare institutions must be well equipped with efficient and effective resources to provide healthcare to a more and more demanding population. As service must meet population needs, and costs with human resources represent a large amount of the operational costs in those institutions, accurate workforce planning is a very important task.

In the healthcare sector, nurses represent the main workforce to plan. The special kind of service they provide is to look after people in different severity conditions and the care cannot be postponed. To determine the nurse staff levels, decision makers must take into account not only the variable demand for nursing care, but also the need to safeguard the health and goodwill of the nurses themselves, beyond the guidelines and legislation in force.

This section continues with a review of studies related to nurse workforce planning in general, and nurse staffing problems, in particular, highlighting some aspects that still deserve research. The definition of the multi-skill nurse staffing problem under study is then presented. Section Methods presents a two-phase methodology and describes the optimization model. An application using real-world data from a Brazilian hospital is described in Section Results, illustrated with the spreadsheet implementation of the model. Besides, a sensitivity analysis is conducted. A discussion of the experimental results follows in the corresponding section. The paper ends with the Section Conclusions.

### Nurse workforce planning

In hospitals that work around the clock every day, the daily nurse work must be organized in shifts, and for each nurse a schedule must be posted in advance, indicating the sequence of days off and daily work shifts for the next planning period. Therefore, hospital administration needs to solve three interrelated problems, as stated in the seminal paper of Warner [1]. At the strategic decision level, the staffing problem consists of determining the dimension of the workforce required to provide the healthcare needed in all hospital units over a long-term planning horizon [2]. In the medium-term, at the tactical level, the scheduling/rostering problem is solved, so as to determine the set of schedules for all nurses of each unit that meets the variable demand per shift during a planning period, typically of one month, or 28 days, while respecting labour agreements [1, 3]. In the short-term, at the operational decision level, the rerostering problem determines the necessary changes in the current roster when daily disruptions occur [4, 5].

These different types of decision have associated models that have been widely studied, however in the majority of the cases to tackle specific situations, as surveyed for human resources in general [6, 7]. In the nurses’ work planning context there are also some surveys revealing the considerable amount of research work in this area, which is mainly due to the specific legislation of each country and specific norms of the institutions [8–10]. Therefore, it is hard to conceive a general system for nurse staffing, scheduling/rostering and rerostering suitable for addressing all cases.

In the current study, we focus on the staffing problem with the purpose of determining the multi-skill nursing staff level per shift, derived from nurse-to-patient indicators applied to the demand for healthcare. We also take into account the further need of assigning nurses to shifts, complying with the labour legislation in force. Adequate nursing care is forced by hard constraints because it is the primary goal, while the minimization of salary costs is set as objective function.

We propose a new model to this problem and, relying on the model, we developed a spreadsheet-based tool that can be easily used in hospital units.

### Nurse staffing

The nurse staffing problem must take into account nurses’ workload measures and, in some cases, the allocation of nurses to the different daily shifts, considering the particular working environment, patient safety ratios and labour rules. Many authors highlight the positive correlation between the decrease of staffing levels and the decrease in quality of nurse care [11–13] as well as with the decrease in financial performance of hospitals [14]. Other authors also stress the negative impact of nurse turnover and the use of temporary nurses on patient care and nurse satisfaction [11, 15–17].

Hence, defining accurate nurse staffing levels is an important objective of every hospital administration, under pressure to reduce costs, while providing high quality health services. Approaches to nurse staff dimensioning are commonly concerned with determining the minimum number of nursing care hours per patient. Hurst [18] classified the methods to measure nursing workload into five categories: (1) professional judgement, based on the experience and knowledge of professionals; (2) average number of nurses per occupied bed; (3) the acuity-quality method, based on patient dependency; (4) regression analysis; and (5) the timed-task/activity method, based on the type and frequency of nursing interventions. Nursing workload naturally depends on the condition of patients, and several scoring systems to determine nurse-to-patient ratios have been proposed [19, 20]. Therefore, hospitals and healthcare public administrations frequently lay down specific instructions to guide hospital managers and head nurses in staffing and scheduling tasks. That is the case of the “Resolução COFEN-293/2004” from the Brazilian Nurse Federal Council, which sets the nursing hours per patient for inpatient units according to four categories of illness severity [21].

Elkhuizen et al. [22] propose a general model that determines multi-shift nurse staff levels. The model does not consider different nursing skills and works for 24-h cover. It calls for computing average bed occupation on a daily basis, using calendar work days. Legal minima for nurse-to-patient ratios, set by international guidelines (USA and Australia), were used to establish the corresponding different ratios for three shifts (early, late and night). These ratios, together with the bed occupation and some coefficients to include absences, led to the advised number of nurses per shift. An application to a real setting is presented along with a what-if analysis. Kortbeek et al. [23] propose stochastic models for nurse staffing per shift with demand based on nurse-to-patient ratios. One of the models simultaneously staffs permanent nurses per hospital unit and floating nurses that may work at any hospital unit. The authors do not consider different nurse skills. They apply their models to a hospital in Amsterdam. To the best of our knowledge, the first work that explicitly models multi-skill multi-shift nurse staffing is that of Bordoloi and Weatherby [24], specifically developed for one hospital unit in Alaska. These authors present a linear programming model considering three skill categories and three shifts of equal length, thus defining nine decision variables. The demand per day (in hours) was estimated from historical data and divided per shifts according to the nurses’ experience. The model minimizes salary costs and considers ratio constraints on work provided by differently-skilled staff and demand constraints per shift. These authors also perform a linear programming sensitivity analysis. More recently, Harper et al. [2] present a comprehensive study for multi-skill multi-shift nurse staffing in United Kingdom hospitals. The model proposed is a stochastic linear programming model that considers the minimization of costs aggregated over one year, subject to demand-fulfilment constraints per day and for different skills. The proposed computational tool is applied with user-defined parameters to obtain an aggregate level of staff.

Other authors integrate staffing and scheduling. Venkataraman and Brusco [25] propose a stochastic methodology that starts by solving a mixed integer linear programming model for a six-month period. This model has two integer variables (number of full-time and part-time nurses) and six continuous variables (overtime hours per month) and its solution is given as input to another mixed integer linear programming model for the scheduling problem. Different shifts and different skills are considered. More recently, Maenhout and Vanhoucke [26] also integrate nurse staffing and scheduling, applied to a hospital in Ghent. This study proposes a mixed integer linear programming model where the staffing decisions are represented by surplus and slack variables that account for under or over staffing of multi-skilled nurses per shift, with respect to the required number of nurses per ward, skill, day and shift.

No research work was found that addresses the nurse staffing problem by simultaneously considering the multi-skill staff levels required for different length shifts; respecting the scores of a scoring system as well as labour legislation, guidelines and internal institution rules; and balancing the number of employees per skill and per shift. Moreover, the literature lacks computational tools to dimension differently-skilled nursing staff, that are easy for practitioners to use, and adaptable to different hospital environments.

### Problem definition

The study reported in this paper concerns a nurse staffing problem that aims to determine the size of nursing teams in a Brazilian hospital context. Personnel are grouped into two categories according to healthcare nursing skills: nurses – the group of the more skilled personnel; and nursing technicians – those who perform the auxiliary and basic care tasks, although professionally certified. In the hospital, apart from this permanent pool, dimensioned at the strategic decision level, there is also a floating pool of nurses and technicians, dimensioned at the tactical level, outside the scope of this study. The number of nurses should be at least a given proportion of the total staff, depending on the patient mix. Once assigned to a shift, nurses and technicians of this pool do not switch to another shift during the long-term planning horizon. Personnel assigned to night shift cannot work on consecutive days, due to labour legislation. Therefore, these personnel are grouped into odd night shift and even night shift workers. Moreover, the staff level in these two shifts must be equal, due to hospital rules.

The main objective of the nurse staffing problem is to determine the number of nurses and of nursing technicians to be assigned to the permanent pool of a hospital unit, complying with the demand for healthcare per shift and minimizing salary costs. The needs for healthcare service must be continuously satisfied by workers who are entitled to a specific number of days off and holidays, as well as absences. In this context, all types of absences are taken into account by the application of a Technical Safety Index (TSI) [21].

## Methods

**Fig. 1:**
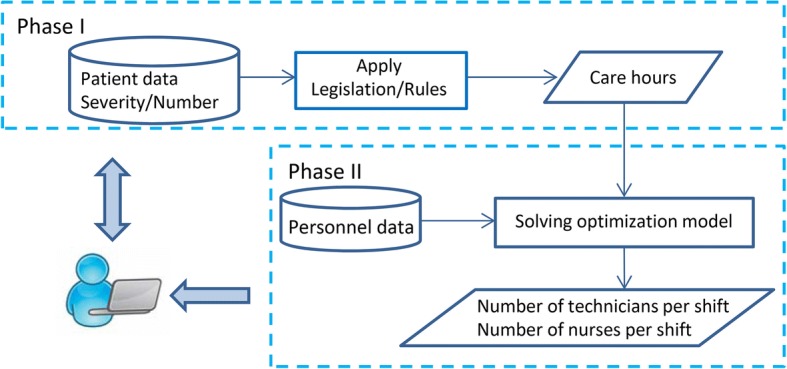
Staffing method

To determine the dimension of the permanent pool of nurses and nursing technicians for a hospital, the proposed methodology follows two phases, as illustrated by Fig. 1. The staffing is solved per hospital unit because each unit has different requirements in terms of nursing work, due to the different patient illness severity. For instance, the Intensive Care Unit (ICU), the Neurosurgery Unit, and the Cerebrovascular Accident Unit (CVA) typically treat high severity illness patients. In addition, the personnel of the permanent pool are assigned per hospital unit. The method considers the demand for a day since the guidelines stipulate the amount of daily nursing care.

### Phase I

*Phase I* computes the required number of nursing care hours per day, according to the specific guidelines in use. For this computation, the necessary data are the estimated number of patients and their respective illness severity. For instance, the Brazilian COFEN guidelines [21] recommend a minimum number of nursing hours per patient, per day for each category of illness severity (minimal, intermediary, semi-intensive, or intensive care). In addition, the minimum ratio of nurses to nursing workers is also computed (as proposed by COFEN).

The output of *Phase I* is given as input to *Phase II*.

### Phase II

*Phase II* proceeds with the dimensioning of personnel, additionally considering the following data from the institutional rules: the hourly salary costs per level of skill (nurses and technicians) and shift (morning, afternoon, odd and even nights), the shifts lengths, lower bounds for the staff per skill and shift, and parameters to balance the staff between shifts and per skill. Other data come from COFEN guidelines: the TSI, acting as a buffer to cover all types of personnel absences, and the ratio of nurses to total staff.

The problem consists of determining the number of nurses and technicians required per shift, respecting the following conditions:

- the total number of hours of nurse and technician work assigned to all shifts cannot be less than the required nursing care hours per day (*hours*) [Constraints (1)];
- the percentage over the minimum staff level required to cover all types of personnel absences must be satisfied, corresponding to the application of the TSI (represented by the parameter *p*) [Constraint (2)];
- the number of nurses must be at least a given percentage-ratio (*prop*) of the total number of nursing-workers, i.e. nurses plus technicians [Constraint (3)];
- the number of workers, of each skill, assigned to morning and afternoon shifts do not differ more than a specified parameter, so to balance the workload among these shifts (represented by parameters *α*_*i*_, *i* = *n*, *t*) [Constraints (4)];
- two groups of night shifts are considered – odd nights (night 1) and even nights (night 2), because staff cannot work on consecutive nights, according to labour legislation [Constraints (5)];
- a minimum staff level per shift for each of nurses and technicians must be respected [Constraints (6)].

Adequate nursing care is enforced by the above hard constraints, while control of costs is achieved through the objective function of the model [Function (7)], where costs of both types of night shift are considered.

An optimization model is proposed and its mathematical formulation is presented in [Additional file 1]. It corresponds to an integer linear programming problem, insofar as it involves optimization of a linear function depending on non-negative integer variables subject to linear (in)equalities that express the constraints of the model. The solution for this model is obtained using a standard optimization solver.

The minimum imposed by Constraints (6) ensures that the demand to be immediately satisfied is covered, while Constraints (1) guarantee that these and other nursing activities, which do not need immediate execution in a given shift, are performed during the day. The minimum staff levels per shift can be adapted to accommodate different requirements for care per shift.

The decision maker can execute *Phase I* followed by *Phase II* repeatedly, with different demand scenarios to extract managerial indicators, thus supporting the decision making process regarding the staffing of the permanent pool. This allows him/her to evaluate the impact on staff level due to fluctuations in demand for healthcare. Complementarily, other constraints imposed by hospital management rules, sector guidelines, and legislation can be considered.

The results of *Phase II* – solution of the staffing problem – will be inputs for scheduling/rostering. In this hospital context, nursing-workers do not change shifts.

## Results

The methodology is illustrated with the case of Hospital São José (HSJ). HSJ is part of Irmandade Santa Casa de Misericórdia de Porto Alegre, Brazil, and its units are characterized by the type of healthcare service provided: Emergency, Inpatient Units (ICU, CVA, PRT/CNV, and Neurosurgery), Intensive Care, Diagnosis and Treatment Units, and Outpatient and Surgical Center [27].

### Experiment description

**Fig. 2:**
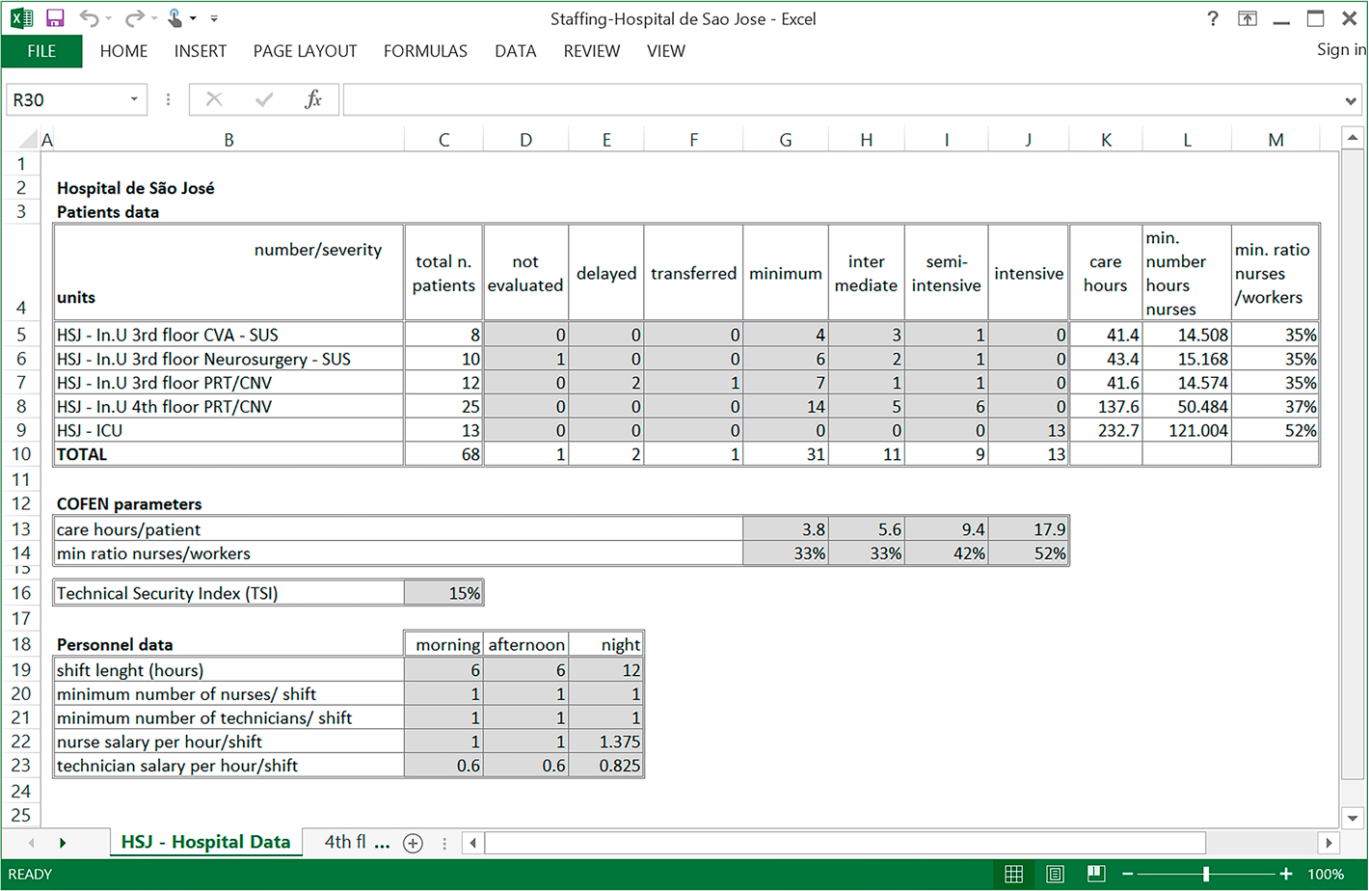
Data used for model application

For the experiment, we considered real data from five HSJ units. Figure 2 displays the data used for model application. For each unit, data concerning the demand of one day was considered, and the following numbers were compiled: number of inpatients requiring minimal care, intermediary care, semi-intensive care and intensive care. The unclassified inpatients were counted as needing intermediary care.

The Brazilian guidelines for nursing professional practice, COFEN [21], define the minimum number of nursing care hours per patient per 24 h (Art. 4th): 3.8 h per patient requiring minimal care, 5.6 h for intermediary care, 9.4 h for semi-intensive care and, 17.9 h for intensive care, respectively. COFEN also specifies a minimum ratio of nurses to the total workforce which is set according to the category with the largest number of inpatients. This proportion is set to 33% for the minimal and intermediary care, 42% for the semi-intensive care, and 52% for the intensive care. In addition, the TSI is set to 15%, according to COFEN.

The method is applied using a Microsoft Excel spreadsheet [28]. The data in spreadsheet “HSJ – Hospital Data” are organized in three tables (Fig. 2). The first table, “Patients data”, contains the number of patients and their corresponding illness severity for the different hospital units on a specific day. The second table, “COFEN parameters”, contains the model’s parameters. Finally, the table “Personnel data” is used in *Phase II*. The shifts’ lengths, which are not all equal, are consistent with the hospital operating rules. The minimum number of nurses and technicians per shift (*lines 20* and *21*) are set to one. Due to confidentiality reasons, the labour costs are omitted, and we assume a labour cost of 1 monetary unit per hour per nurse, and that the labour cost for technicians is 60% of that value. Work in the night shift receives an increase of 37.5% in salary.

The output of *Phase I*, for each unit, is in column “care hours” and is given by summing up the product of the number of patients in the unit (*columns G* to *J*) by the required care hours per patient severity (*line 13*), and also in *column* *M*, the ratio of nurses with respect to the total of nursing-workers.

### Spreadsheet implementation of the model

**Fig. 3:**
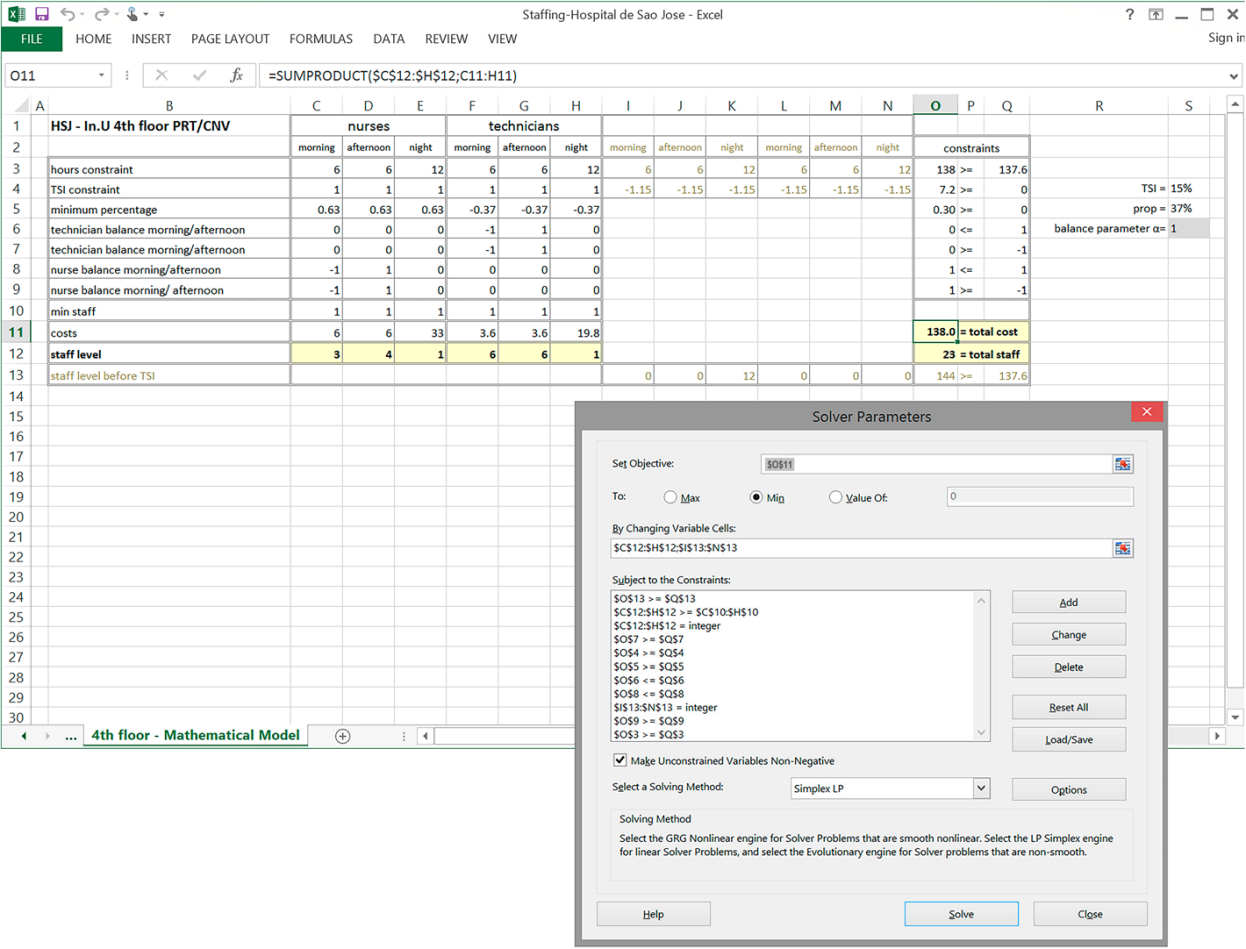
Data and model results for Hospital São José, unit “In. U 4^th^ floor PRT/CNV”

The model for *Phase II* was implemented using the Solver from Excel [28] as shown in Fig. 3 for the unit “In. U 4^th^ floor PRT/CNV”. The spreadsheet “4^th^ floor – Mathematical Model” is organized into four main areas including all data, variables and formulae of the mathematical model in [Additional file 1].

The “nurses” area comprises *columns C* to *E* which include parameters (*lines 3* to *11*) and variables (*line 12*). *Line 3*, “hours constraint”, contains the shifts lengths (in this case equal for all shifts and skills) taken from sheet “HSJ – Hospital Data”. *Line 5* “minimum percentage” contains the parameters needed for Constraint (3) of the problem by setting *prop* equal to the value in *cell S5*, which is equal to the minimum ratio of nurses to workers for this unit, in *cell M8* of “HSJ – Hospital Data” sheet (Fig. 2). *Line 10* contains the minimum staff level in each shift which, in this case, is set equal to 1 in Constraints (6) for all shifts. *Line 11* has the salary costs per shift, which are computed by multiplying the value of labour cost per hour by the shift length in hours, using the data in spreadsheet “HSJ – Hospital Data”. As the model in the solver just refers to a single day, to take into account the staff for the two types of night shift, “night 1” and “night 2”, the cost for the night shift is counted twice (Constraints (5)). *Line 12* is reserved for the variables’ values. For “technicians”, the corresponding information is given in *columns F* to *H*.

*Columns I* to *N* include data for auxiliary variables to respect staff levels before TSI application, which are used in Constraints (1) and (2): *line 3* contains the shifts length to be included in the first inequalities of Constraints (1); *line 4* implements Constraint (2), with TSI from “HSJ – Hospital Data”, and *line 13* contains the values of these variables.

Finally, *columns O* to *Q* include information for solving the problem: constraints and the objective function. *Column O* (from *lines* 3 to 9) contains the excel formulae to be used in Solver, while *column Q* contains the constant terms related to Constraints (1) to (4).

To balance the number of nurses and technicians between morning and afternoon shifts, a single parameter is set by the user in *cell S6*. In this application, we consider the balance parameter to be equal both for nurses and technicians.

The mathematical model is formulated for Solver software, in the window “Solver Parameters”: in the field “Set Objective” *cell O11* must be inserted. The respective value is the objective function value to be minimized through the variables in *C12:H12* and *I13:N13*. Constraints are added in the constraints section, all variables are set to nonnegative values and the method selected is “Simplex LP” (a simplex based branch-and-bound algorithm).

By clicking button “Solve”, the algorithm runs and determines the model output, which is also shown in Fig. 3. The solution obtained, in *cells C12* to *H12*, indicates the number of nurses and technicians for the three daily shifts. In this case, the solution gives 3 nurses for the morning, 4 for the afternoon and 1 for the night shifts; for technicians the numbers are 6, 6 and 1, respectively. The total salary cost for this solution is 138.0 monetary units, as shown in *cell O11*.

### Sensitivity of the model to parameter variation

To assess the sensitivity of the model solutions regarding variations of the parameters, we analysed the impact of changes in the minimum staff level per shift along with variations of the balance parameter (*α*_*n*_ = *α*_*t*_). Table 1 presents the results of this analysis.

**Table 1:** Impact of changes in parameters

parameters		staff level for nurses					staff level for technicians					total staff	total cost
min. staff	balance *α*_*n*_ = *α*_*t*_	morning	afternoon	night 1	night 2	total	mor-ning	afternoon	night 1	night 2	total		
1	1	3	4	1	1	9	6	6	1	1	14	23	138.0
1	2	4	3	1	1	9	5	7	1	1	14	23	138.0
1	3	2	5	1	1	9	5	7	1	1	14	23	138.0
2	1	2	3	2	2	9	5	5	2	2	14	23	171.6
2	2	2	3	2	2	9	4	6	2	2	14	23	171.6
2	3	2	3	2	2	9	4	6	2	2	14	23	171.6
3	1	3	3	3	3	12	3	3	3	3	12	24	216.0
3	2	3	3	3	3	12	3	3	3	3	12	24	216.0
3	3	3	3	3	3	12	3	3	3	3	12	24	216.0

Increasing the minimum staff level required per shift increases the total cost because it forces the assignment of extra staff to more expensive shifts. Increasing the balance parameter provides more flexibility to distribute workers among shifts, and consequently the cost decreases. For instance, a solution for balance parameter *α*_*n*_ = 2, requiring a maximum difference of 2 nurses between the morning and afternoon shifts, is also a solution when *α*_*n*_ = 3 which requires a maximum difference of 3 workers between the morning and afternoon shifts, while a solution for *α*_*n*_ = 3 may not satisfy a balance parameter of *α*_*n*_ = 2. This can be observed in Table 1, when minimum staff is 1, the solution for *α*_*n*_ = 2 includes 4 nurses in the morning and 3 in the afternoon, also satisfying *α*_*n*_ = 3; while the solution for *α*_*n*_ = 3 considers 2 nurses in the morning and 5 in the afternoon, and thus does not satisfy *α*_*n*_ = 2.

When the minimum number of staff is fixed, variations of the balance parameter value do not have an impact on the total cost or total number of staff. In fact, when the minimum number of staff is fixed to 1, different solutions with the same cost were obtained for different values of the balance parameter (from 1 to 3). When the minimum staff level is set to 2, the total cost increases by 24.2% and the staff levels remain equal to 9 for nurses and 14 for technicians. Increasing the minimum staff level from 2 to 3 increases the total cost by 25.8%, leading to a solution where at least 12 nurses and 12 technicians must be assigned, which sum up to 24 (3 workers of each skill and shift).

## Discussion

The methodology proposed was applied to data related with bed occupation and type of nursing care required by patients in several units of HSJ. In particular, a study was undertaken for the unit “In. U 4^th^ floor PRT/CNV”, for a duration of eight consecutive days, from Wednesday, September 7, 2016. The first part of Table 2 displays the data relating to the number of patients by illness severity level, the number of nursing care hours according to COFEN (as explained in subsection “Hospital data”) and the corresponding minimum ratio of nurses to workers (*prop* parameter in the model). The second part of the table presents the results obtained from *Phase II* of the method.

**Table 2:** Real data and model results for Hospital São José, in unit “In. U 4^th^ floor PRT/CNV”

date	weekday	data								results of *Phase I*		
		number of patients by illness severity level								care hours COFEN	minimum ratio nurses/workers COFEN (%)	
		total		minimal	intermediary		semi-intensive	intensive				
07–09-2016	Wednesday	34		21	10		3	0		164.0	35	
08–09-2016	Thursday	25		15	8		2	0		120.6	34	
09–09-2016	Friday	26		16	8		2	0		124.4	34	
10–09-2016	Saturday	21		13	7		1	0		98.0	34	
11–09-2016	Sunday	23		15	6		2	0		109.4	35	
12–09-2016	Monday	25		17	7		1	0		113.2	34	
13–09-2016	Tuesday	34		24	8		2	0		154.8	34	
14–09-2016	Wednesday	35		22	11		2	0		164.0	34	
staff levels												
date	results of *Phase II*										real situation	
	number of nurses per shift				number of technicians per shift				number of nurses	number of technicians	number of nurses	number of technicians
	morning	afternoon	night 1	night 2	morning	afternoon	night 1	night 2				
07–09-2016	3	4	2	2	7	8	1	1	11	17	4	22
08–09-2016	3	3	1	1	5	6	1	1	8	13	4	22
09–09-2016	3	3	1	1	5	6	1	1	8	13	4	22
10–09-2016	2	2	2	2	3	4	1	1	8	9	4	22
11–09-2016	2	3	2	2	4	5	1	1	9	11	4	22
12–09-2016	2	3	1	1	5	5	1	1	7	12	4	22
13–09-2016	4	4	1	1	7	7	1	1	10	16	4	22
14–09-2016	3	4	2	2	7	8	1	1	11	17	4	22

The results show that total staff required varies between 17 and 28 workers. The total staff in the real situation is constant and equal to 26. This number of workers is greater than or equal to the staff levels obtained by the method for all days except for Wednesdays, for which two additional workers are needed. Regarding the number of nurses, the results reveal that in the real situation there is a lack of nurses if the COFEN guidelines are applied, while the number of technicians is greater than the one obtained by the model. This reveals that in the current real situation the minimum ratio of nurses to the total workforce, which depends on the mix of patients’ illness severity (subsection “Hospital data”) is not covered by the permanent pool of nurses. In these cases, nurses from the floating pool may be assigned.

As a result of this experiment, we may observe that the proposed methodology was conceived to support the hospital management decision making process, so to improve the personnel dimensioning, and better agree with the COFEN guidelines.

## Conclusions

A new methodology was developed to determine the multi-skill nursing staff level to be assigned per shift during a long-term planning horizon, in a hospital, complying with legislation, nurse work guidelines, and rules in force, while minimizing hospital costs. This complex staff dimensioning problem was adapted to the Brazilian context, considering a real world hospital, with two skill categories – nurses and technicians – and three work shifts of different durations. The proposed method applies to a single unit and allows the hospital management to determine the staff level per shift and per skill category as a function of the mix of severity. The workforce for a hospital can be obtained by executing the method for all its units.

The staff dimensioning method, at the strategic decision level, will provide input for lower level decision stages concerning personnel planning.

Another contribution of this study is a decision support tool, embedding the staffing method and implemented in a Microsoft Excel spreadsheet. [Additional file 2] contains the tool described in this manuscript. Most managers are familiar with usage of this software, therefore, the spreadsheet model is designed in an intelligible way to allow for an easy utilization. The tool also provides support to analyse scenarios with different mix of severity. Moreover, the decision maker can modify different parameters, such as shift lengths, salary costs, minimum staff level required per shift and skill, and the parameter to balance workforce, and evaluate their impact on results. The model and the tool developed are easily customizable to integrate the working rules of different hospital contexts.

As future work we intend to apply and evaluate the developed methodology in other healthcare contexts, namely in Portuguese public hospital units.

## Additional files

Additional file 1: Optimization model. The mathematical model used to represent the problem and to implement in the computational tool. (PDF 193 kb)

Additional file 2: Computational tool. The computational tool to support decision making on multi-skill nurse staffing in hospital units. (XLSX 19 kb)

## Abbreviations

CVA: Cerebrovascular Accident Unit
HSJ: Hospital São José
ICU: Intensive Care Unit
PRT/CNV: Unit for Patients with Health Plan
SUS: Unified Health System (in Brazil)
