# Supplementary material for: A computational application for multi-skill nurse staffing in hospital units
Source: BMC Med Inform Decis Mak. 2018 Jun 28;18:53. doi: 10.1186/s12911-018-0638-2 (PMC6025742; doi:10.1186/s12911-018-0638-2)
Supplement: Supplementary file 1 — Optimization model. The mathematical model used to represent the problem and to implement in the computational tool. (PDF 193 kb) [file 12911_2018_638_MOESM1_ESM.pdf]

## Optimization Model

The optimization model used for staffing purposes in *Phase II* of the method considers a previously chosen hospital unit and a specific day. The hospital nursing staff includes two different skill levels, indexed by $n$ for nurses and $t$ for nursing technicians. Daily work is divided into three shifts: morning (*M*), afternoon (*A*) and night ($N^1$ – "night 1" for an odd day night shift and $N^2 -$ "night 2" for an even day night shift). The set of shift types is represented by $S = \{M, A, N^1\}$ considering without loss of generality an odd day.

The following parameters are known for the specific hospital unit:

$h_{is}$ – duration of shift *s* for skill *i* workers (in hours);

$hours$ – demand of nursing service in the specified day (care hours);

$p$ – TSI parameter;

$prop$ – minimum ratio of nurses to the total workforce;

$min_{is}$ – minimum staff of skill *i* necessary for shift *s*;

$c_i$ – cost per worker of skill *i* per hour;

$\bar{c}$ – percentual extra cost per hour during the night period (from 10 pm to 7 am);

$c_{is}$ – shift cost per worker of skill *i* assigned to shift *s*, where $c_{is} = c_i h_{is}$, for *s* = $M$, $A$, and $c_{is} = c_i h_{is}(1 + \bar{c})$, for $s = N^1, N^2$;

$\alpha_i$ – parameter to balance the staff of skill $i$ between shifts ($i = n, t$).

Considering the integer variables:

$x_{is}$ – number of workers of skill $i$ necessary for shift $s$ to cover the demand of the specific day ($i = n, t$; $s \in S$), auxiliary variables;

$\tilde{x}_{is}$ – number of workers of skill $i$ necessary for shift $s$ to cover the demand of the specific day and taking into account the $p$% requested by TSI ($i = n, t; s = M, A, N^1, N^2$);

the constraints of the proposed integer linear programming model follow:

$$\sum_{s\in S}\sum_{i=n,t} h_{is}\, x_{is} \geq hours;\ \sum_{s\in S}\sum_{i=n,t} h_{is}\, \tilde{x}_{is} \geq hours \tag{1}$$

$$\sum_{s\in S}\sum_{i=n,t} \tilde{x}_{is} \geq (1+p/100)\ \sum_{s\in S}\sum_{i=n,t} x_{is} \tag{2}$$

$$\sum_{s\in S} \tilde{x}_{ns} \geq prop\ \sum_{s\in S}\sum_{i=n,t} \tilde{x}_{is} \tag{3}$$

$$\tilde{x}_{iM} \leq \tilde{x}_{iA} + \alpha_i;\ \tilde{x}_{iM} \geq \tilde{x}_{iA} - \alpha_i; i = n, t \tag{4}$$

$$\tilde{x}_{iN^2} = \tilde{x}_{iN^1}; i = n, t \tag{5}$$

$$\begin{cases} \tilde{x}_{is} \geq min_{is};\ s \in S; i = n, t \\ \tilde{x}_{is} \text{ non-negative integer; } s = M, A, N^1, N^2; i = n, t \\ x_{is} \text{ non-negative integer; } s \in S; i = n, t. \end{cases} \tag{6}$$

The objective function representing total salary costs is formulated as follows:

$$\min z = \sum_{s=M,A,N^1,N^2}\sum_{i=n,t} c_{is}\, \tilde{x}_{is}. \tag{7}$$

The solution of this problem, through the values of variables $\tilde{x}$, provides the number of nursing personnel of the two skills per shift for the hospital unit. Variables $\tilde{x}_{iN^2}$ for the even night shift are set equal to $\tilde{x}_{iN^1}$ by Constraints (5) and are not included in the model except in the objective Function (7).
